# *QuickStats:* Age-Adjusted Percentage* of Adults Aged ≥18 Years Who Reported Their Level of Satisfaction with Life,^†^ by Disability Status^§^ — National Health Interview Survey,^¶^ United States, 2022

**DOI:** 10.15585/mmwr.mm7251a5

**Published:** 2023-12-22

**Figure:**
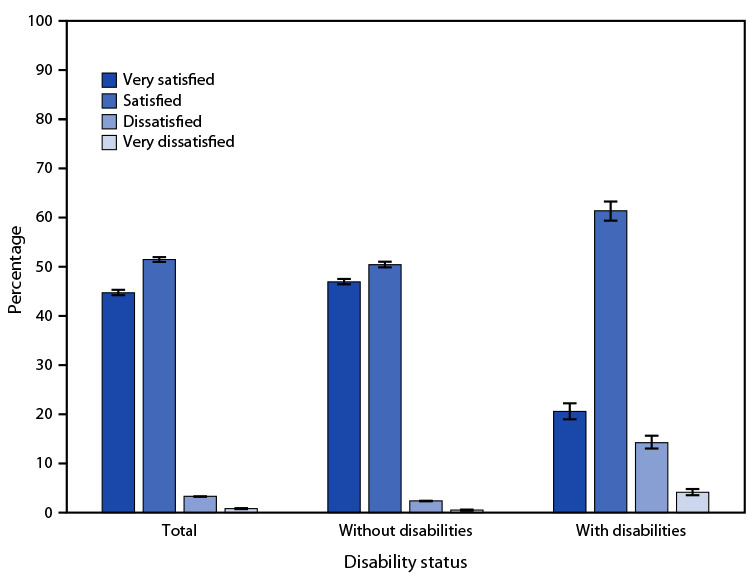
In 2022, 44.6% of adults aged ≥18 years reported they were very satisfied with their life, 51.3% reported they were satisfied, 3.3% reported they were dissatisfied, and 0.8% reported they were very dissatisfied. Adults without disabilities were more likely to be very satisfied (46.8%) or satisfied (50.3%) with their life than dissatisfied (2.4%) or very dissatisfied (0.5%). Adults with disabilities were more likely to be satisfied with their life (61.2%) compared with very satisfied (20.5%), dissatisfied (14.2%), or very dissatisfied (4.1%). Adults without disabilities were more likely than adults with disabilities to be very satisfied with their life. Conversely, adults with disabilities were more likely than adults without disabilities to be satisfied, dissatisfied, or very dissatisfied.

**Source:** National Center for Health Statistics, National Health Interview Survey, 2022. https://www.cdc.gov/nchs/nhis/index.htm

For more information on this topic, CDC recommends the following link: https://www.cdc.gov/ncbddd/disabilityandhealth/index.html

